# Organoid intelligence for developmental neurotoxicity testing

**DOI:** 10.3389/fncel.2024.1480845

**Published:** 2024-10-08

**Authors:** Dowlette-Mary Alam El Din, Jeongwon Shin, Alexandra Lysinger, Matthew J. Roos, Erik C. Johnson, Timothy J. Shafer, Thomas Hartung, Lena Smirnova

**Affiliations:** ^1^Center for Alternatives to Animal Testing, Department of Environmental Health and Engineering, Bloomberg School of Public Health, Johns Hopkins University, Baltimore, MD, United States; ^2^Department of Biomedical Engineering, Johns Hopkins University, Baltimore, MD, United States; ^3^Research and Exploratory Development Department, Johns Hopkins University Applied Physics Laboratory, Laurel, MD, United States; ^4^Center for Computational Toxicology and Exposure, Office of Research and Development, U.S. Environmental Protection Agency, Research Triangle Park, NC, United States; ^5^Center for Alternatives to Animal Testing Europe, University of Konstanz, Konstanz, Germany; ^6^Doerenkamp-Zbinden Chair for Evidence-based Toxicology, Baltimore, MD, United States

**Keywords:** organoid, organoid intelligence, developmental neurotoxicity, artificial intelligence, machine learning, synaptic plasiticity

## Abstract

The increasing prevalence of neurodevelopmental disorders has highlighted the need for improved testing methods to determine developmental neurotoxicity (DNT) hazard for thousands of chemicals. This paper proposes the integration of organoid intelligence (OI); leveraging brain organoids to study neuroplasticity *in vitro,* into the DNT testing paradigm. OI brings a new approach to measure the impacts of xenobiotics on plasticity mechanisms – a critical biological process that is not adequately covered in current DNT *in vitro* assays. Finally, the integration of artificial intelligence (AI) techniques will further facilitate the analysis of complex brain organoid data to study these plasticity mechanisms.

## 1 Introduction

Neurodevelopmental disorders – classified by deficits in developmental domains including motor skills, social interactions, language acquisition, and cognition (Hirvikoski et al., 2021) – show a rising trend in diagnoses (Straub et al., 2022; CDC, 2022; CDC, 2024). The increasing surge in cases cannot be solely attributed to genetic factors or alterations in diagnostic criteria (Tran and Miyake, 2017; Hansen et al., 2015; King and Bearman, 2009). Environmental exposures, including developmental neurotoxicants, have emerged as potential contributing factors to the increasing prevalence of neurodevelopmental disorders (Carlsson et al., 2021).

Efforts to understand the interaction between environmental exposures and neurodevelopment in higher throughput and more human-relevant models have led to the creation of the developmental neurotoxicity (DNT) *in vitro* battery (IVB) (OECD, 2023). The DNT IVB is a series of *in vitro* assays using both human and rodent cells that measures a variety of key neurodevelopmental processes (OECD, 2023). While the DNT IVB addresses most aspects of neurodevelopment on the molecular and cellular levels, it does not have an assay to measure brain function such as neuroplasticity, using human cells.

Brain organoids, derived from pluripotent stem cells, recapitulate brain cellular composition and functionality and thus are an emerging tool to study neurological disorders as well as brain development and homeostasis in general. Organoid intelligence (OI) is the use of brain organoids as a tool for modeling the neural responses that contribute to higher level functions (such as learning and memory) *in vitro* (Smirnova et al., 2023). As brain organoids contain the important neuronal and glial cell types, mimic aspects of normal neuronal circuitry, and roughly resemble human brain cytoarchitecture (Sandoval et al., 2024; Qian et al., 2016; Paşca et al., 2015; Lancaster et al., 2013; Kadoshima et al., 2013), OI represents a promising tool for DNT testing. OI assays that can measure neuroplasticity (as neuroplasticity mechanisms are essential to learning and memory) (Mateos-Aparicio and Rodríguez-Moreno, 2019) include open- and closed-loop assays and can complement the DNT-IVB when used to assess toxicant impact on plasticity. In open-loop experiments, organoids are provided with information in the form of electrical, chemical, or optogenetic stimuli and their responses are measured in the form of network dynamics changes, which then can be classified so that the input can be predicted based on the measured output. In the closed-loop experiments, feedback is provided to the organoid based on the response to the stimuli, which then is repeated in cycles to record the organoid’s capability to “learn” and modify its output (Smirnova et al., 2023). These assays can quantify the impact of xenobiotics on plasticity mechanisms and information processing. By leveraging the potential of OI, researchers can enhance the detection of compounds with DNT hazard by covering an aspect of neurobiology that is not well captured by current *in vitro* assays.

By utilizing the responses of brain organoids to electrical/chemical/optogenetic stimulation, OI offers a biologically relevant approach to studying neuroplasticity *in vitro*. Overall, this paper examines the potential of OI to enhance current methods, the integration of machine learning (ML) and artificial intelligence (AI) into OI assay development and data analysis, and considerations for scaling up these assays to meet regulatory standards for inclusion in the DNT IVB. Ultimately, this paper advocates for the integration and utility of OI in DNT testing, by creating new *in vitro* assays aimed at functional endpoints in neurotoxicology research that are currently underrepresented.

## 2 Developmental neurotoxicity testing

Neurodevelopmental disorders, which include Autism Spectrum Disorder (ASD) and Attention Deficit/Hyperactivity Disorder (ADHD), are heterogeneous and are classified by impairments in the acquisition of skills in different developmental domains including motor, social, language, and cognition (Hirvikoski et al., 2021). The prevalence of neurodevelopmental disorders has been increasing over time, with one in four publicly- and one in nine privately-insured children diagnosed with one or more neurodevelopmental disorders by eight years of age (Straub et al., 2022). In addition, it was shown from 2016–2019 that six million children aged 3–17 had ADHD (CDC, 2022), while in 2020 one in 36 children in the USA was diagnosed with ASD (CDC, 2024). The Autism and Developmental Disabilities Monitoring Network showed that the prevalence of ASD for 8 year olds increased over 121% over 10 years from 2002 to 2012 (Christensen et al., 2016; Prevalence of Autism Spectrum Disorders, 2002). Lastly, ADHD prevalence in children aged 3 to 17 increased over 25% between 2003 to 2015 (Centers for Disease Control and Prevention (CDC), 2010; National Center for Health Statistics, United States, Centers for Disease Control and Prevention, 2015). This increase in neurodevelopmental disorders cannot be attributed to genetic factors, diagnostic criteria, and reporting methods alone (Tran and Miyake, 2017; Hansen et al., 2015; King and Bearman, 2009). Determining the cause(s) of neurodevelopmental disorders remains a challenge due to the complexity of these diseases, but previous research has documented that exposure to environmental toxicants can negatively impact neurodevelopment (Carlsson et al., 2021; Grandjean and Landrigan, 2014; Landrigan, 2010). There are current Organization for Economic Co-operation and Development (OECD) and Environmental Protection Agency *in vivo* testing guidelines for DNT (OECD, 2007; U.S. Environmental Protection Agency, 1998). However, testing for DNT is not routinely required for all compounds and for pesticides, it is typically only conducted when signs of neurotoxicity are observed in other required studies or the compound is structurally related to known neurotoxicants. As a result, many compounds have never been tested for their DNT potential (Behl et al., 2019; Smirnova et al., 2014). In addition, it is challenging to test all the newly registered compounds due to the limitations of the current testing paradigm. This paradigm, based on animal models, is expensive (Tsuji and Crofton, 2012; Rovida and Hartung, 2009; Paparella et al., 2020), time-consuming (1–2 years) (Tsuji and Crofton, 2012; Rovida and Hartung, 2009; Paparella et al., 2020), low-throughput (Paparella et al., 2020), raise 3Rs conflicts (Paparella et al., 2020), and is not always physiologically relevant (Behl et al., 2019; Smirnova et al., 2014; Blum et al., 2023). As a result, chemical safety testing is transitioning away from *in vivo* animal tests and instead moving toward using *in vitro* testing and integrated approaches to testing and assessment (OECD, 2023). This has led to the development of new approach methodologies (NAMs) to study DNT (Bal-Price et al., 2018b; Masjosthusmann et al., 2020; US EPA, 2020). NAMs include *in vitro* and *in silico* methods to inform safety assessment (Stucki et al., 2022) and present the opportunity for less expensive, high-throughput approaches to address the gaps in toxicity testing. Since an individual NAM in isolation cannot model all aspects of neurodevelopment, a battery of DNT tests was proposed to study key neurodevelopment processes independently of one another (Blum et al., 2023). The *in vitro* battery (IVB) for DNT includes several assays that are meant to characterize the physiological effects of chemicals on the developing neural system including stages of neuronal development. These tests are used as a high-throughput, cost effective method for screening environmental chemicals for DNT (Bal-Price et al., 2018b; Bal-Price et al., 2018a). The DNT IVB can evaluate how a compound impacts different stages of neuronal development, but a human-based *in vitro* assay to determine the impact of compounds on neuroplasticity has not yet been incorporated into the DNT IVB (Bal-Price et al., 2018b; Juberg et al., 2023). As a complement to the DNT IVB, zebrafish assays can be used for behavioral assessment (Bal-Price et al., 2018b). Therefore, there is a need to develop an assay using human cells to include in the DNT IVB to assess neuroplasticity as these mechanisms can help inform changes on the behavioral level.

The standard approach to studying the effects of a chemical or disease on cognitive functions, such as learning and memory, involves utilizing either an animal model or conducting epidemiological assessments with humans. Although epidemiological studies offer insightful data, they have several limitations. These include the observational rather than mechanistic nature of collected data, the constraint on the range of research questions that can be explored due to their focus on an exposed population, and the fact that they are time consuming with limited types of samples that can be collected. In addition, there are challenges with documenting the exact exposure, including its duration and amount, which are difficult to establish, and assignment of effects to a single entity is often difficult due to co-exposures. This makes it difficult to investigate the underlying processes by which cognition is impaired. There is a history of using animal models to evaluate cognitive functions, and there is an array of assays that have been developed and used to measure cognitive impairments. Some animal cognition/behavior assays that exist include the Morris Water Maze, Y-Maze, Novel Object Recognition, Barnes Maze, Radial Arm Maze, Step-through Passive Avoidance, and Reversal Learning tests (d’Isa and Gerlai, 2023; Shaw and Schmelz, 2017). In all animal behavior tests, there are three main elements: (1) a motivating factor; (2) the observed behavior, and (3) the quantified outcome (d’Isa and Gerlai, 2023). These tests can cause stress to the animal or even brief pain through the delivery of mild electrical shocks (d’Isa and Gerlai, 2023). In addition to the ethical concerns, there are many critical challenges associated with the cross-translational research of studying cognition in animals including reproducibility, standardization, and clinical heterogeneity (d’Isa and Gerlai, 2023). Moreover, animal models present a challenge for toxicity testing because they are costly and time consuming. This reduces the throughput of scientific studies, thereby limiting the number of chemicals, windows of exposure, and doses that can be tested. Due to all these limitations, there is a need for a human cell-based *in vitro* assay that can be used to measure the biology underlying cognitive functions in a faster, more cost-effective manner. These gaps represent an opportunity for OI plasticity assays to be used for *in vitro* DNT testing, which can subsequently reduce the requirement for resource-intensive animal-based learning and memory tests.

## 3 OI for DNT

Throughout human brain development, the brain is highly susceptible to exposures (Bick and Nelson, 2016; Miguel et al., 2019). Brain development is a regulated process that involves neural stem cell proliferation, differentiation, neuronal migration, synaptogenesis, neuronal circuit development, synaptic maturation and pruning, myelination and gliagenesis (National Academies Press, 2000; Jiang and Nardelli, 2016; Tau and Peterson, 2010; Zhou et al., 2024). Throughout development, the brain continues to modulate its neural circuit connectivity (Jiang and Nardelli, 2016). As cognition is increasingly understood through neuronal circuitry, the proper development of these circuits is critical for cognitive development (Tau and Peterson, 2010; Zhou et al., 2024). Exposure to xenobiotics during brain development could significantly impair cognition and overall cognitive functions later in life (Bick and Nelson, 2016; Miguel et al., 2019; Zhou et al., 2024).

Toward understanding the impact of diverse exposures on brain development, efforts have been directed toward generating *in vitro* models aimed at replicating these cellular processes within controlled environments (Cao, 2022; Fan et al., 2022; Matsui and Shinozawa, 2021; Vashishat et al., 2024; Yang et al., 2022). Microphysiological systems (MPS) is an umbrella term, covering different types of advanced cultures, including organoids, spheroids, microfluidics, and organ-on-chip technology (Barreras et al., 2023). These models serve to elucidate the mechanisms and potential adverse outcomes associated with such exposures (Cao, 2022; Fan et al., 2022; Matsui and Shinozawa, 2021; Barreras et al., 2023). Human brain microphysiological systems (bMPS) are human cell-derived advanced cell cultures, recapitulating key aspects of brain architecture and functionality (Barreras et al., 2023). Among these, induced pluripotent stem cell (iPSC) derived three-dimensional (3D) brain models, such as organoids, spheroids, and assembloids, have shown more complexity than traditional two-dimensional (2D) monolayer cultures. Brain organoids can recapitulate the correct cellular composition, including neuronal and glial cell types through self-organization and co-differentiation (Barreras et al., 2023; Andersen et al., 2020; Anderson et al., 2021; Birey et al., 2017; Kim and Chang, 2023; Pamies et al., 2017; Qian et al., 2019; Quadrato et al., 2017; Sloan et al., 2017). They also exhibit cytoarchitecture (Kim and Chang, 2023), network connectivity (Sharf et al., 2022), and aspects of electrical activity found in the developing brain (Qian et al., 2016; Paşca et al., 2015; Birey et al., 2017). The 3D cytoarchitecture in bMPS models provides increased cell–cell interactions, helping regulate key aspects of neuronal network development including proliferation, differentiation, and migration, which directly impacts neuronal network function (Yang et al., 2022; Aschner et al., 2017). While 2D models are useful, they lack 3D cytoarchitecture and have demonstrated lower neuronal activity compared to organoid models (Trujillo et al., 2019), which plays a critical role in neuroplasticity processes (Citri and Malenka, 2008).

Among the many methods to generate bMPS, two common approaches are unguided and guided differentiation, which generate neural or region-specific organoids, respectively (Qian et al., 2019). Region-specific organoids, such as the forebrain (Qian et al., 2016), midbrain (Qian et al., 2016; Jo et al., 2016), hypothalamus (Qian et al., 2016), cerebral (Paşca et al., 2015; Lancaster et al., 2013; Citri and Malenka, 2008; Jo et al., 2016), and hippocampal (Pomeshchik et al., 2020; Jacob et al., 2020), are just some of the models that have been developed so far. Although organoids are able to recapitulate important features of human brain development, it is important to note that they are not as complex as the human brain (Qian et al., 2019), and there is variability across different organoid generation protocols (Qian et al., 2019). However, they are simplified models of the human brain that provide an opportunity to study the cellular mechanisms underlying brain development and functionality and how xenobiotics could influence those functions. Utilizing these region-specific organoids individually can help to better understand brain-region specific impacts of xenobiotics on function. Furthermore, combining these different brain regions using assembloids or microfluidic techniques could potentially provide greater model complexity. Assembloids have been shown to produce more complex oscillatory activity compared to individual organoids (Osaki et al., 2024). In addition, recent work demonstrates that combining organoids of different brain regions, such as thalamic and cortical organoids, to study synaptic plasticity in a simplified human neural circuit is capable of both short- and long-term plasticity (Patton et al., 2024). While no model can replicate the full complexity of the human brain, these organoid and assembloids-based approaches offer powerful tools for studying the toxicological effect of xenobiotics on brain development and function.

In recent developments, the term organoid intelligence (OI) has been proposed as the use of organoids as a promising model for studying the neural responses that contribute to cognitive functions such as learning and memory *in vitro* (Smirnova et al., 2023). While current studies with organoids have not demonstrated the capabilities of learning or memory, they have exhibited both short (Zafeiriou et al., 2020; Cai et al., 2023) and long-term synaptic plasticity (Patton et al., 2024). These processes are fundamental for learning and memory (Whitlock et al., 2006). Therefore studying these mechanisms can provide insight into brain function and studying their disruption following chemical exposure could provide information about neurotoxicity hazard.

## 4 How OI can be used for Developmental Neurotoxicity testing?

**Figure 1:**
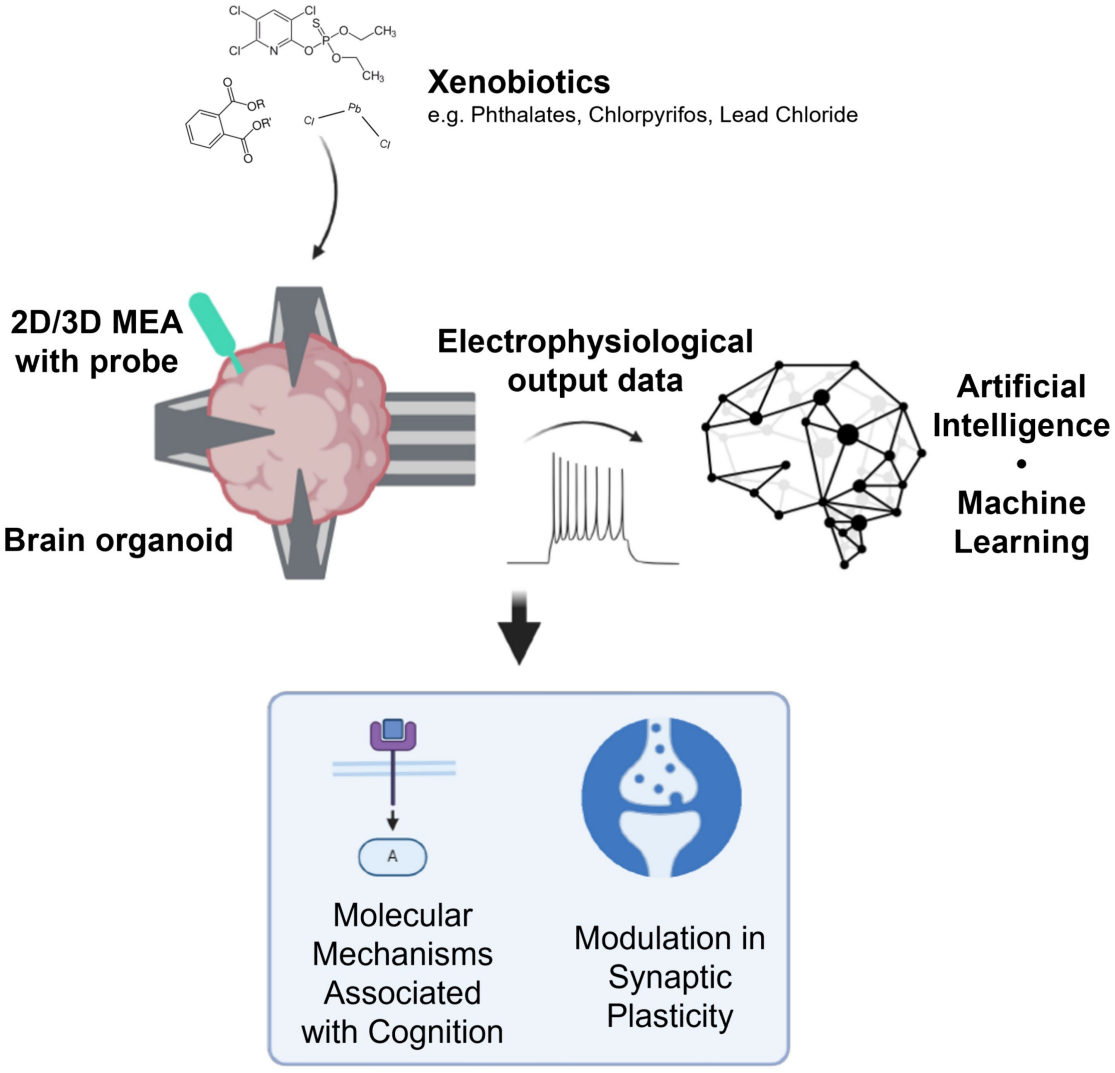
Outline of organoid intelligence for developmental neurotoxicity testing. Using brain organoids interfacing with tools such as high-density microelectrode arrays (HD-MEAs) and optogenetic probes, electrophysiological data can be used to inform how xenobiotics impact the molecular mechanisms associated with cognition and modulate synaptic plasticity. Created in BioRender. Alam el din, D. (2024) BioRender.com/g91a488 (this is the publication license).

OI can complement and enhance the current DNT IVB, expanding the functional aspects of the battery by including tests for neuroplasticity. Taking the current battery of tests to a new level, OI offers the opportunity to quantify the impact of xenobiotics on brain organoid information processing at different stages of brain organoid development. Specifically, it enables a focused analysis of neuronal responses to information, identifying compounds that perturb these functions and potentially shedding light on the mechanisms by which xenobiotics impair neuroplasticity in the developing brain (Figure 1).

While OI is a new frontier and assays are still being developed, there is already promising data to support its utility for developmental neurotoxicity testing. Organoids have been shown to exhibit long-term plasticity using standard patch clamping techniques (Patton et al., 2024) and exhibit long-term network changes after stimulation on microelectrode arrays (MEAs) (Zafeiriou et al., 2020). In addition, brain organoids completed reservoir computing tasks, which involved predicting nonlinear dynamics and identifying sensory signals (Cai et al., 2023). Moreover, dissociated rat cortical cells cultured in 2D on MEAs altered their neural networks in response to repetitive electrical training stimuli and demonstrated their ability to distinguish individual signals when exposed to multiple stimuli (Isomura et al., 2015). These tasks showcase how *in vitro* models including organoids can be used to study biological information processing and network plasticity, resembling unsupervised machine learning. These tests could be considered open-loop assays, as they stimulate and record electrophysiology data from the organoids without task-related feedback.

In addition, while not an organoid, 2D *in vitro* neuronal networks have been shown to successfully “learn” how to play a variant of the game “pong” in a simulated game environment through real-time closed-loop electrophysiological stimulation and recording on a high-density MEA (HD-MEA) (Kagan et al., 2022). Furthermore, other 2D *in vitro* neuronal network models were able to successfully navigate a virtual robot through an arena with obstacles (Tessadori and Chiappalone, 2015), move a robotic arm (Chapin et al., 1999), and “learn” using a closed-loop experimental design (Shahaf and Marom, 2001; Li et al., 2007; le Feber et al., 2010; Pimashkin et al., 2013; Sinapayen et al., 2017). These experiments represent the successful implementation of closed-loop *in vitro* experiments that can be applied to organoids and used for OI.

**Figure 2:**
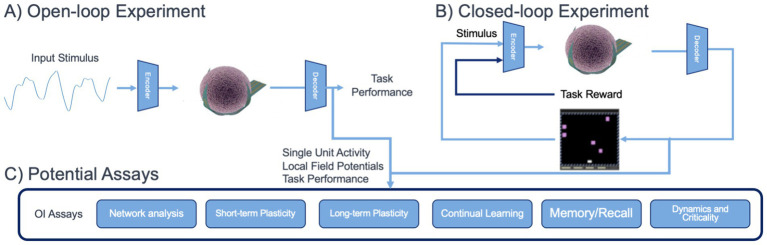
Envisioned use of OI for *in vitro* assessment assays for brain organoids. **(A)** An open-loop experiment, mimicking sensory processing, where task feedback is not used to stimulate the organoid. **(B)** A closed-loop experiment, mimicking reinforcement learning or behavioral tasks. **(C)** Potential assays which can be derived from brain organoid physiological data collected during open- and closed-loop experiments.

By utilizing both open- and closed-loop experimental paradigms as shown in Figure 2, OI could provide detailed information as to how xenobiotics potentially modulate neuroplasticity across different stages of brain organoid development. It could also quantify changes in information processing in neural circuits and criticality of neural dynamics, both valuable metrics for studying brain function that could inform neurotoxic hazard potential.

### 4.1 Integration of AI for analysis and interpretation of open- and closed-loop experiments

HD-MEAs and rapidly maturing novel electrode technologies such as 3D shell MEAs (Huang et al., 2022), 3D-self-rolled biosensor arrays (Kalmykov et al., 2021), nanowires (Li et al., 2019), mesh electrodes (McDonald et al., 2023; Park et al., 2021), and high density probe electrodes (Steinmetz et al., 2021), allow for signals to be presented to and recorded from organoids, in some cases using hundreds to thousands of channels (Steinmetz et al., 2021; Miccoli et al., 2019; Müller et al., 2015). The complexity and volume of this data pose a challenge, but not an insurmountable one, as the advancements in AI over the past decade now provide suitable computational hardware and techniques for analyzing high-dimensional organoid signals (Pachitariu et al., 2016). OI for DNT testing can leverage extensive advances in ML approaches for *in vivo* and *in vitro* neuroscience, allowing for automated analysis and computational modeling of neural systems at a similar scale.

#### 4.1.1 Capabilities and limitations of current machine learning and AI approaches for OI

Moving forward, ML and AI algorithms will facilitate the detection, analysis, and interpretation of structured activity patterns that emerge in developing brain organoids. In general, OI for DNT can benefit from emerging approaches for AI for Science (Wang et al., 2023), which are advancing in many disciplines. Neuroscience, in particular, has lagged behind other domains, such as genomics or material science, when applying ML and AI tools for data analysis. There is now, however, rapid progress in the area (Johnson et al., 2023). ML and AI algorithms relevant to OI for DNT testing include many tools for automating analysis of functional neuroscience data. This includes established algorithms for identifying individual units in high dimensional electrophysiology recordings (Pachitariu et al., 2016), processing regions of interest to extract traces in calcium imaging data (Giovannucci et al., 2019), and estimating functional connectivity of *in vitro* networks (Puppo et al., 2021). These established approaches can greatly improve the throughput and scalability of OI experiments and are well validated using existing *in vivo* and *in vitro* models at the scale of envisioned OI experiments (thousands of individual electrodes over many hours of recording). Ultimately, these approaches are expected to be central to a set of OI assays for functional analysis as shown in Figure 2.

In addition to accelerating computational analysis, AI and ML approaches are being applied to additional aspects of exploratory data analysis, visualization, and interpretation. While such approaches are more nascent, they hold great promise for giving interpretable insight for OI experiments to explore DNT. In the broader neuroscience domain, such methods are already being widely applied to *in vivo* animal recordings (Le and Shlizerman, 2022; Schneider et al., 2023) to discover lower-dimensional activity embeddings, determine the most exciting stimuli in a closed-loop experiment (Walker et al., 2019), and decode variables related to sensory and motor processing (Pei et al., 2022). In addition, there are extensive packages, such as the Brain Modeling Toolkit (Dai et al., 2020), for creating computational models of neural activity and plasticity, which can be used for prediction as well as numerical experiments. Such methods are equally applicable to brain organoid electrophysiology and functional recordings. It is critical, however, to recognize the limitations of many emerging ML and AI algorithms and modeling approaches, including a lack of generalization to new datasets. Unique OI preparations for DNT experiments may require preliminary experimental validation, as well as collecting and labeling new data for fine tuning of algorithms.

#### 4.1.2 OI experiments and validation of computational AI and ML models

A critical aspect of integrating AI and ML approaches for analysis of OI data is establishing experimental paradigms, such as outlined in Figure 2. ML and AI approaches for automating steps of analysis, such as spike sorting, functional connectivity estimation, region of interest identification, and computation of key metrics and statistics can be used in both closed and open-loop experiments. In these cases, pre-trained models can be deployed to rapidly ingest experimental data and create secondary data products. Integration of more complex AI tools will require validation against experimental data.

Validation of experimental approaches and AI software tools can leverage existing data archives and standards from the neuroscience community. Examples of this include the DANDI archive, which contains extensive *in vivo* recordings from mammalian nervous systems in a standardized Neurodata Without Borders (Rübel et al., 2022) format. These data can be used to create validation datasets to ensure OI data produce raw data and secondary data products of sufficient quality for further analysis. In addition, experimental approaches can be constructed to explicitly compare dynamics, plasticity, and activity across OI and *in vivo* systems. In addition, the community should endeavor to create data analysis challenges focused AI and ML models for prediction and interpretation of neural data, following the model of successful *in vivo* benchmark datasets (Pei et al., 2022). In addition to *in vivo* benchmarks, the community should create artificial agent benchmarks which can be used to provide performance targets for OI experimental tasks and frameworks (Khajehnejad et al., 2024).

In addition, using experimental data gathered during closed-loop experiments, AI algorithms can be trained to model sophisticated learning rules in organoids. These rules include either phenomenological rules, rules built on neuroscience principles such as Hebbian learning and spike-timing dependent plasticity, or rules built on neuroscience learning theories such as predictive coding (Huang and Rao, 2011; Rao and Ballard, 1999) or the free-energy principle (Friston, 2010). As with open-loop experiments, progression of organoid learning can be monitored to assess the impact of xenobiotics. These can be assessed using tasks derived from AI algorithm development (Staley et al., 2021) as well as simulations of behavioral experiments conducted *in vivo* (Molano-Mazon et al., 2022). As the field develops, such closed-loop experiments may play the role of established behavioral experimental paradigms, relating toxicity mechanisms to functional activity changes to behavioral outcomes. Careful design of validation experiments will be required, including hold out experimental conditions, to ensure robust and reproducible model fits.

Challenges remain, however, and the nature of any given organoid system should be considered with respect to its relationship to human brains. In particular, current single-region brain organoids lack the brain macrostructure and thus both the organoids and AI models of those organoids are unlikely to be adequate models of certain types of learning, such as those that leverage delayed reward (O’Doherty et al., 2003) or relate to complementary learning theories (McClelland and Goddard, 1996). Scaling is also a challenge; if organoids are to be used in screening, then systems will need sufficient throughput (multi-well plate format, which is challenging for more advanced MEA devices or more complex perfused organoid systems) to test multiple chemicals simultaneously. In addition, variability in electrode positioning and interfacing with organoid’s neurons may create difficulties in experimental reproducibility. Advances in both organoid and interface consistency are likely necessary to ensure consistent results across organoids, time, and neurotoxicity testing protocols.

### 4.2 Regulation and adoption of OI for developmental neurotoxicity testing

To have an impact on assessment of environmental chemicals for DNT, the OI field will need to evolve from its current state as a tool for basic neurobiology to become a useful tool for applied toxicology. This will require several advancements and transformations. These approaches will have to be more widely available and adopted by the toxicology community, which will require the availability of training and thus specialists, which have expertise in the use of OI and its application to different types of problems. The requisite equipment and biological models will also need to be widely available. It will take some time for this to happen, but as it was seen for gene editing and genomics approaches, this approach can become more mainstream. As part of this process, approaches will need to become more standardized and variability of data produced by these approaches will need to be quantified and shown to be comparable to or better than data obtained from animal models (Paparella et al., 2020). In the near future, OI approaches could be incorporated into DNT hazard assessment as part of a tiered approach. Thomas et al. (2019) have outlined a temporally tiered approach to chemical testing where transcriptomic approaches comprise a 1st tier, the current assays in the DNT-IVB comprise a 2nd tier, and organoid and OI approaches could become a viable 3rd tier. As the technology improves and allows for higher throughput approaches (for example, closed -loop experiments in multi-well MEA plates), then these approaches can be employed in earlier tiers (e.g., 1st or 2nd) for DNT screening and characterization. By following the approach taken for assays in the current DNT-IVB (testing common sets of chemicals, making data publicly available, development of criterion for acceptability of data), OI-derived assays could eventually be incorporated into the battery. Regardless of what tier these approaches might contribute to now or in the future, regulatory acceptance will be facilitated by the development of case-studies that demonstrate the ability of organoids and OI to inform regulatory decision-making.

## 5 Conclusion

In conclusion, incorporating OI into DNT testing represents a promising tool for advancing toxicology research and improving regulatory approaches. By addressing the limitations of the current testing paradigm, OI offers a human based approach to study neuroplasticity *in vitro*. In addition, the integration of AI further enhances the analysis of complex brain organoid data, providing information on the impact of xenobiotics on neurodevelopment. Overall, OI is a promising new approach that has the potential to be used to study neurotoxicity mechanisms, contribute to higher throughput chemical assessments, and aid in regulatory decisions.

## Data Availability

The original contributions presented in the study are included in the article/supplementary material, further inquiries can be directed to the corresponding author.
